# Pancreatitis of biliary origin, optimal timing of cholecystectomy (PONCHO trial): study protocol for a randomized controlled trial

**DOI:** 10.1186/1745-6215-13-225

**Published:** 2012-11-26

**Authors:** Stefan A Bouwense, Marc G Besselink, Sandra van Brunschot, Olaf J Bakker, Hjalmar C van Santvoort, Nicolien J Schepers, Marja A Boermeester, Thomas L Bollen, Koop Bosscha, Menno A Brink, Marco J Bruno, Esther C Consten, Cornelis H Dejong, Peter van Duijvendijk, Casper H van Eijck, Jos J Gerritsen, Harry van Goor, Joos Heisterkamp, Ignace H de Hingh, Philip M Kruyt, I Quintus Molenaar, Vincent B Nieuwenhuijs, Camiel Rosman, Alexander F Schaapherder, Joris J Scheepers, Marcel BW Spanier, Robin Timmer, Bas L Weusten, Ben J Witteman, Bert van Ramshorst, Hein G Gooszen, Djamila Boerma

**Affiliations:** 1 Department of OR/Evidence Based Surgery, Radboud University Nijmegen Medical Centre, HP 690, PO 9101, Nijmegen, HB 6500, the Netherlands; 2 Department of Surgery, University Medical Center Utrecht, HP G04.228, PO 85500, Utrecht, GA 3508, the Netherlands; 3 Department of Surgery, St Antonius Hospital, PO 2500, Nieuwegein EM 3430, the Netherlands; 4 Department of Surgery, Academic Medical Center, PO 22660, Amsterdam, DD 1100, the Netherlands; 5 Department of Radiology, St Antonius Hospital, PO 2500, Nieuwegein, EM 3430, the Netherlands; 6 Department of Surgery, Jeroen Bosch Hospital, PO 90153, Den Bosch, ME 5200, the Netherlands; 7 Department of Gastroenterology, Meander Medical Center, PO 1502, Amersfoort, BM 3800, the Netherlands; 8 Department of Gastroenterology, Erasmus Medical Center, PO 2040, Rotterdam, CA 3000, the Netherlands; 9 Department of Surgery, Meander Medical Center, PO 1502, Amersfoort, BM 3800, the Netherlands; 10 Department of Surgery, Maastricht University Medical Center and NUTRIM School for Nutrition, Toxicology and Metabolism, PO 5800, Maastricht, AZ 6202, the Netherlands; 11 Department of Surgery, Gelre Hospital, PO 9014, Apeldoorn, DS 7300, the Netherlands; 12 Department of Surgery, Erasmus Medical Center, PO 2040, Rotterdam, CA 3000, the Netherlands; 13 Department of Surgery, Medisch Spectrum Twente, PO 50000, Enschede, KA 7500, the Netherlands; 14 Department of Surgery, Radboud University Nijmegen Medical Centre, HP 690, PO 9101, Nijmegen, HB 6500, the Netherlands; 15 Department of Surgery, St. Elisabeth Hospital, PO 90151, Tilburg, LC 5000, the Netherlands; 16 Department of Surgery, Catharina Hospital, PO 1350, Eindhoven, EJ 5623, the Netherlands; 17 Department of Surgery, Hospital Gelderse Vallei Ede, PO 9025, Ede, HN 6710, the Netherlands; 18 Department of Surgery, University Medical Center Groningen, PO 30001, Groningen, RB 9700, the Netherlands; 19 Department of Surgery, Canisius-Wilhelmina Hospital, PO 9015, Nijmegen, GS 6500, the Netherlands; 20 Department of Surgery, Leiden University Medical Center, PO 9600, Leiden, RC 2300, the Netherlands; 21 Department of Surgery, Reinier de Graaf Gasthuis, PO 5011, Delft, AD 2625, the Netherlands; 22 Department of Gastroenterology, Rijnstate Hospital, PO 9555, Arnhem, TA 6800, the Netherlands; 23 Department of Gastroenterology, St Antonius Hospital, PO 2500, Nieuwegein, EM 3430, the Netherlands; 24 Department of Gastroenterology, Hospital Gelderse Vallei Ede, PO 9025, Ede, HN 6710, the Netherlands

**Keywords:** Acute pancreatitis, Gallstones, Trial, Common bile duct, Cholecystitis, Endoscopic retrograde cholangiopancreaticography, Surgery, Cholecystectomy, Timing, Mortality

## Abstract

**Background:**

After an initial attack of biliary pancreatitis, cholecystectomy minimizes the risk of recurrent biliary pancreatitis and other gallstone-related complications. Guidelines advocate performing cholecystectomy within 2 to 4 weeks after discharge for mild biliary pancreatitis. During this waiting period, the patient is at risk of recurrent biliary events. In current clinical practice, surgeons usually postpone cholecystectomy for 6 weeks due to a perceived risk of a more difficult dissection in the early days following pancreatitis and for logistical reasons. We hypothesize that early laparoscopic cholecystectomy minimizes the risk of recurrent biliary pancreatitis or other complications of gallstone disease in patients with mild biliary pancreatitis without increasing the difficulty of dissection and the surgical complication rate compared with interval laparoscopic cholecystectomy.

**Methods/Design:**

PONCHO is a randomized controlled, parallel-group, assessor-blinded, superiority multicenter trial. Patients are randomly allocated to undergo early laparoscopic cholecystectomy, within 72 hours after randomization, or interval laparoscopic cholecystectomy, 25 to 30 days after randomization. During a 30-month period, 266 patients will be enrolled from 18 hospitals of the Dutch Pancreatitis Study Group. The primary endpoint is a composite endpoint of mortality and acute re-admissions for biliary events (that is, recurrent biliary pancreatitis, acute cholecystitis, symptomatic/obstructive choledocholithiasis requiring endoscopic retrograde cholangiopancreaticography including cholangitis (with/without endoscopic sphincterotomy), and uncomplicated biliary colics) occurring within 6 months following randomization. Secondary endpoints include the individual endpoints of the composite endpoint, surgical and other complications, technical difficulty of cholecystectomy and costs.

**Discussion:**

The PONCHO trial is designed to show that early laparoscopic cholecystectomy (within 72 hours) reduces the combined endpoint of mortality and re-admissions for biliary events as compared with interval laparoscopic cholecystectomy (between 25 and 30 days) after recovery of a first episode of mild biliary pancreatitis.

**Trial registration:**

Current Controlled Trials: ISRCTN72764151

## Background

Acute pancreatitis is a major healthcare problem. The disease is the third most common gastrointestinal reason for acute hospital admission, carrying a mortality rate of 5% with a total annual cost of $2.2 billion in the USA alone [1,2]. In most western countries approximately 30 to 55% of cases are caused by gallstones or sludge, referred to as biliary pancreatitis [3]. After biliary pancreatitis, patients may experience a recurrent episode of biliary pancreatitis or other biliary events, such as acute cholecystitis, common bile duct obstruction, cholangitis or biliary colics [4,5]. In order to prevent these recurrent biliary events, international guidelines advise performing cholecystectomy or endoscopic sphincterotomy (ES) after biliary pancreatitis [6,7]. Failure to provide definitive treatment exposes the patient to (potentially fatal) risks of biliary diseases [8].

The timing of cholecystectomy in patients with clinically severe pancreatitis, with local complications such as pancreatic necrosis and organ failure, is deliberately delayed until local complications have resolved, typically after some 6 weeks [9].

In patients with mild pancreatitis, international guidelines advise cholecystectomy directly after recovery or in the first 2 to 4 weeks after discharge for mild biliary pancreatitis [4,6,7,10,11]. However, there is no consensus on the ideal moment of cholecystectomy. Audits from Germany, the UK, the USA and Italy report that the majority of specialists perform an interval cholecystectomy 6 to 12 weeks after discharge, due to uncertainty about the efficacy and safety of an early cholecystectomy and for logistical reasons [12-15]. In a nationwide study in the Netherlands, we demonstrated that three-quarters of the patients admitted with mild biliary pancreatitis underwent cholecystectomy a median of 6 weeks after discharge [16]. Comparable results were found in a systematic review by our group where over 50% of patients underwent cholecystectomy at least 6 weeks after discharge [17].

Several nonrandomized studies in patients with mild biliary pancreatitis suggested that early cholecystectomy, as compared with interval cholecystectomy, prevents recurrent biliary pancreatitis or other complications of gallstone disease, without adding risks due to a more difficult surgical dissection in the early days following pancreatitis [16,18]. Because the current literature may be flawed by selection bias, a randomized study is needed to confirm that early cholecystectomy is indeed both superior to interval cholecystectomy and safe. The aim of this study is to investigate whether early laparoscopic cholecystectomy, within 72 hours after recovery of a first episode of mild biliary pancreatitis, as compared with interval laparoscopic cholecystectomy, 25 to 30 days after recovery, reduces mortality and acute re-admissions for biliary events.

## Methods/Design

### Design

The PONCHO trial is a randomized controlled, parallel-group, assessor-blinded, superiority multicenter trial. Patients will be randomly allocated to receive early laparoscopic cholecystectomy, within 72 hours after randomization, or interval laparoscopic cholecystectomy, 25 to 30 days after randomization.

### Primary endpoint

The primary endpoint is a composite endpoint of mortality and acute re-admissions for biliary events (that is, recurrent biliary pancreatitis, acute cholecystitis, symptomatic/obstructive choledocholithiasis requiring endoscopic retrograde cholangiopancreaticography (ERCP) including cholangitis (with/without ES), and uncomplicated biliary colics; see Table 1 for definitions) occurring within 6 months following randomization.

**Table 1:** Primary endpoint: definitions of biliary events

**Biliary event**	**Definition**
Biliary pancreatitis	Diagnosis of acute pancreatitis if at least two of the three following features are present [19]:
	1. Upper abdominal pain;
	2. Serum lipase or amylase levels above three times the upper level of normal;
	3. Characteristic findings of acute pancreatitis on cross-sectional abdominal imaging.
	Biliary pancreatitis if one of the following definitions is present [20]:
	1. Gallstones and/or sludge diagnosed on imaging (transabdominal or endoscopic ultrasound or computed tomography);
	2. In the absence of gallstones and/or sludge, a dilated common bile duct on ultrasound (>8 mm in patients ≤75 years old or >10 mm in patients >75 years old);
	3. The following laboratory abnormality: alanine aminotransferase (ALAT) level >2 times higher than normal values, with ALAT >aspartate aminotransferase.
Acute cholecystitis	Defined according to the 2007 Tokyo classification, grade I to III [21,22].
	A. Local signs of inflammation:
	1) Murphy’s sign;
	2) RUQ mass/pain/tenderness.
	B. Systemic signs of inflammation:
	1) Fever;
	2) Elevated C-reactive protein;
	3) Elevated white blood cell count.
	C. Imaging findings characteristic of acute cholecystitis
	Definite diagnosis
	1) One item in A and one item in B are positive;
	2) C confirms the diagnosis when acute cholecystitis is suspected clinically.
Biliary colic	Upper abdominal pain (either right upper quadrant or epigastric pain) lasting at least 30 minutes, according to the Rome criteria [22].

### Secondary endpoints

We hypothesize that early laparoscopic cholecystectomy is both effective and safe and therefore included the following secondary endpoints to assess such an effect: individual endpoints of the composite endpoint; cholangitis (Table 2); number of biliary colics registered in patient diary; difficulty of cholecystectomy (scored by visual analog scale 0 to 10; 5 being averagely difficult); conversion to open cholecystectomy (measure of technical difficulty); total length of hospital stay; need for ICU admission and total length of ICU stay; and total direct and indirect costs.

**Table 2:** Secondary endpoint: definitions

**Biliary event**	**Definition**
Cholangitis	All of the following features (as previously defined) [20]:
	1) Serum total bilirubin level >40 μmol/l (>2.3 mg/dl) and/or dilated common bile duct (>6 mm) on transabdominal or endoscopic ultrasound or computed tomography;
	2) Temperature >38.5°C.
Organ failure	Failure of one or more of the following organ systems [19]:
	1) Respiratory: PaO_2_ ≤60 mmHg or need for mechanical ventilation;
	2) Cardiovascular: systolic blood pressure <90 mmHg or need for catecholamine support;
	3) Renal: creatinine level >177 μmol/l after rehydration or need for hemofiltration or hemodialysis (not including pre-existent renal failure).
Biliary leakage	Defined according to the Amsterdam criteria [23]:
	Type A: cystic duct leaks or leakage from aberrant or peripheral hepatic radicals;
	Type B: major bile duct leaks with or without concomitant biliary strictures;
	Type C: bile duct strictures without bile leakage;
	Type D: complete transection of the duct with or without excision of some portion of the bile duct.

### Safety endpoints

The safety endpoints are: biliary leakage, Amsterdam types A to D (Table 2) [23]; need for additional surgical, radiological or endoscopic interventions [23]; other complications requiring treatment (that is, bacteremia or pneumonia); bleeding requiring reoperation or blood transfusion; and new-onset organ failure (Table 2).

### Study population

**Figure 1:**
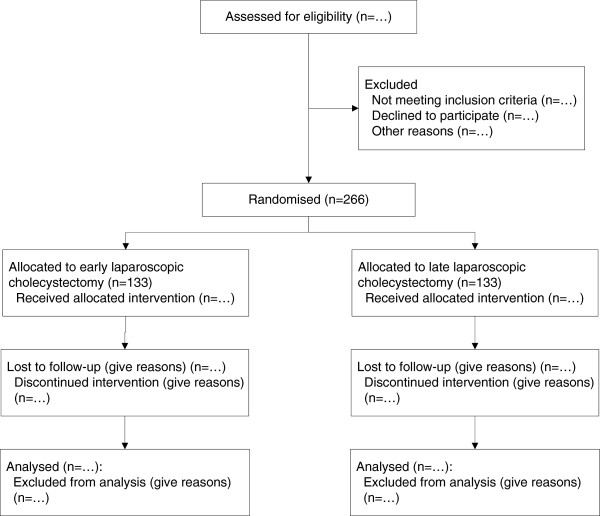
**Flow of participants in the PONCHO trial. **According to CONSORT [24].

All adult patients admitted with a first episode of biliary acute pancreatitis to one of the 18 participating hospitals of the Dutch Pancreatitis Study Group (listed in Authors’ information) will be assessed for eligibility during their hospital admission. Potentially eligible patients are followed until eligibility is established 1 or 2 days before discharge from the hospital. If patients are classified as having mild biliary pancreatitis and fulfill all inclusion and exclusion criteria they are randomized (with a 1:1 ratio and stratified for hospital and ES) to undergo early laparoscopic cholecystectomy or interval laparoscopic cholecystectomy (Figure 1).

### Inclusion criteria

Inclusion criteria are: age ≥18 years; diagnosis of acute pancreatitis (at least two of the three following features present [19]: upper abdominal pain; serum lipase or amylase levels above three times the upper level of normal; and characteristic findings of acute pancreatitis on cross-sectional abdominal imaging); mild pancreatitis (fulfilling both [19]: no pancreatic necrosis and/or peripancreatic collections; and no persistent (> 48 hours) organ failure (Table 2)); and biliary pancreatitis (any of the following three definitions [20,25-29]: gallstones and/or sludge diagnosed on imaging (transabdominal or endoscopic ultrasound or computed tomography); in the absence of gallstones and/or sludge, a dilated common bile duct on ultrasound (>8 mm in patients ≤75 years old or >10 mm in patients >75 years old); and alanine aminotransferase level >2 times higher than normal values, with serum alanine aminotransferase levels >aspartate aminotransferase level); and written informed consent.

### Exclusion criteria

Exclusion criteria are: American Society of Anesthesiologists (ASA) III patients >75 years old; ASA IV and V patients; patients with ongoing alcohol abuse or chronic pancreatitis (males >3 units per day, females >2 units per day) [30]; or pregnancy.

### Time of randomization

After eligibility has been confirmed and written informed consent has been obtained, randomization will take place when the following five discharge criteria are met: the treating physician anticipates that the patient can be discharged within 1 or 2 days; no need for opioid analgesics; declining C-reactive protein levels and <100 mg/l; no evidence of local or systemic complications (for example, no fever); and the patient has resumed oral intake.

### Randomization

Randomization is possible 24 hours per day, 7 days per week and is performed centrally by the study coordinator using an Internet randomization module (Clinical Research Unit, Academic Medical Center, Amsterdam, the Netherlands). Randomization is stratified according to whether ES is performed and according to hospital.

Randomization is balanced for ES because ES has a protective effect on the occurrence of recurrent biliary pancreatitis and common bile duct obstruction [31]. This allows for subgroup analysis with or without ES. Randomization is balanced per hospital as treatment strategies, such as the use of intraoperative cholangiography, indication for enteral feeding and endoscopic sphincterotomy, may differ between hospitals.

Permuted-block randomization with varying block size is used and block size is concealed to all investigators involved in this study. Using this method it is impossible for investigators to predict the allocation of trial participants. Concealing the allocation for investigators or trial participants is unfeasible due to the nature of this trial, because trial participants need to be scheduled for an early or interval cholecystectomy.

### Treatment protocol

**Figure 2:**
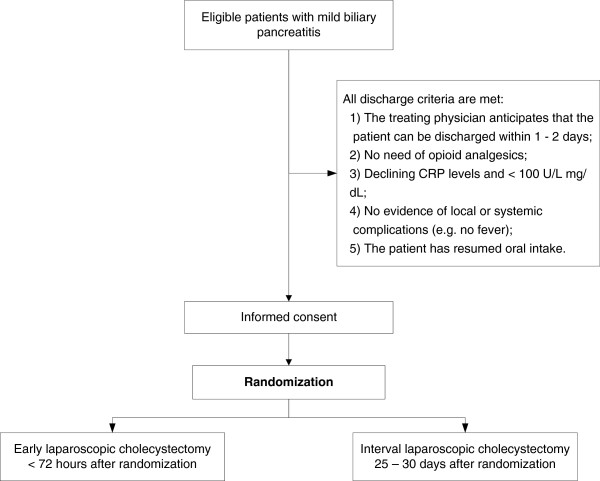
PONCHO overview of eligibility and group allocation.

Group A received early laparoscopic cholecystectomy, within 72 hours after randomization (Figure 2).

As can be seen in the time of randomization criteria, patients will be randomized 1 or 2 days prior to discharge in an attempt to prevent prolonged hospital stay due to a waiting time for cholecystectomy.

Group B received interval laparoscopic cholecystectomy, 25 to 30 days after randomization (Figure 2).

### Surgical details and quality control

Laparoscopic cholecystectomy will be performed according to the guidelines of the Dutch Society of Surgery, which include the critical view of safety technique [32-34]. Patients are operated on by, or under the direct supervision of, a laparoscopically trained surgeon with >100 laparoscopic procedures performed in the previous 5 years. There has to be supervision during the entire procedure, from incision to skin closure.

Single port cholecystectomy will not be performed in this study because there is currently limited experience with this procedure in the Netherlands. If the treating physician decides to perform a primary open cholecystectomy (laparotomy or minilaparotomy), this is allowed in the trial.

Centers may only participate if they intend to randomize at least five patients in the study and are able to perform cholecystectomy <72 hours after randomization.

Given the excellent availability of ERCP/ES in the Netherlands and the lack of experience with intraoperative cholangiography, it is not mandatory to perform an intraoperative cholangiography and, if possible, transcystic stone extraction. In centers with sufficient experience, this technique is allowed. However, laparoscopic choledochal incision and exploration is not allowed as there is insufficient experience with this technique and it is likely to be associated with more morbidity than ERCP/ES. The following data regarding intraoperative cholangiography will be collected: incidence of choledocholithiasis, percentage stones retrieved transcystically, percentage bile duct injury, additional time required, and perceived difficulty (visual analog scale 0 to 10, 5 being averagely difficult).

### Diagnosing and treating biliary pancreatitis

For imaging, in the first 24 hours of admission all patients will undergo an abdominal ultrasound aimed at detecting gallstones and/or sludge in the gallbladder and at determining the diameter of the common bile duct.

Contrast-enhanced computed tomography (CECT) is only performed if the diagnosis for acute pancreatitis is unclear. CECT performed in the first 72 hours of acute pancreatitis cannot rule out necrotizing pancreatitis. Because PONCHO is a pragmatic trial and in current clinical practice CECT is not performed routinely in these patients, CECT is not scheduled for study purposes. CECT will be performed whenever dictated by clinical circumstances based on the judgment of the treating physicians. When the patient’s condition does not improve after 1 week of hospital admission, CECT is performed to visualize the pancreas and abdomen.

ERCP/ES is performed when indicated by the treating physician.

Ursodeoxycholic acid therapy is not recommended after discharge since a recent Dutch randomized multicenter trial showed that this treatment is not effective in preventing recurrent colics and complications [35].

### Data collection and follow-up

There will be telephone follow-up contacts at 3 and 6 months after randomization. When patients are re-admitted to the hospital they are requested to contact the study coordinator. Patients are further asked to register biliary colics during the follow-up period in a gallstone diary. At 6 and 12 weeks after randomization, patients will be asked to complete the Health and Labour questionnaire [36]. Clinical data with regard to baseline characteristics and outcomes will be collected during hospital admission using a case record form. The case record form will be filled out by the local treating physicians, the study coordinator or the study nurse. The study coordinator and the study nurse are allowed to correct wrongly entered data (such as miscalculated patient age or miscalculated disease severity scores). The case record forms will be checked with source data. Only study group personnel will have access to the unblinded source data.

### Safety

An independent Data and Safety Monitoring Committee (DSMC) will evaluate the progress of the trial and will examine safety variables. Every 30 patients, individualized patient description charts including unblinded safety parameters will be presented to the DSMC. In addition to the safety endpoints, death and (serious) adverse events, the incidence of biliary leakage, Amsterdam type D [23], will be of special interest. Extra monitoring of this severe complication is clinically important. The incidence of bile duct injury (Amsterdam types A to D) has a range between 0.16 and 1.5% in the Netherlands [21].

After full explanation of the data presented by the investigators, the members of the DSMC will discuss the consequences of the data presented in the absence of the investigators. Adverse events are defined as ‘any undesirable experience occurring to a subject during a clinical trial, whether or not considered related to the intervention’ (that is, early cholecystectomy). All involved physicians will repetitively be asked to report any potential adverse events. These adverse events will be listed and discussed by the DSMC. The outcome of the meeting of the DSMC will then be discussed with the trial steering committee. The outcome will also be sent to the Institutional Review Board of the Radboud University Nijmegen Medical Centre. All possible severe adverse events will be reported to the Central Committee on Research involving Human Subjects (Centrale Commissie Mensgebonden Onderzoek) and the Institutional Review Board using the online module [37].

### Ethics

The study will be performed in accordance with the declaration of Helsinki and the Dutch Wet Medisch-wetenschappelijk Onderzoek met Mensen (Medical Research Involving Human Subjects Act). The Institutional Review Board of the Radboud University Nijmegen Medical Centre approved the protocol on 22 July 2010. Secondary approval was sought from all local ethics committees. Informed consent will be obtained from each participating patient in oral and written form prior to randomization. The PONCHO trial is registered in the ISRCTN register with identification number ISRCTN72764151.

After approval of the protocol, three amendments were approved by the ethics board. These amendments followed new regulations in the Netherlands for the reporting of (serious) adverse events and minor protocol changes. The content of the amendments are incorporated in this protocol.

### Statistical aspects

#### Sample size calculation

The PONCHO trial is a superiority trial, hypothesizing a reduction in the primary endpoint in favor of early cholecystectomy compared with the current practice of interval cholecystectomy. Data from our nationwide retrospective analysis on timing of cholecystectomy after mild biliary pancreatitis have been used to calculate the sample size [16,38]. In this previous analysis, 6.0% (*n* = 15) of 249 patients with mild biliary pancreatitis were re-admitted within 4 weeks after discharge for recurrent biliary problems prior to cholecystectomy, including 5% recurrent biliary pancreatitis. Because this study was retrospective it might have missed several recurrent complications (arbitrarily set at 20% missed endpoints: 0.2×6% = 1.2%). Furthermore, some 1% of patients may be re-admitted after cholecystectomy for recurrent biliary events. This amounts to an expected incidence of the primary endpoint in the interval laparoscopic cholecystectomy group of 8.2% (6.0 + 1.2 + 1.0). This number is quite conservative compared with the 10% re-admission rate within 4 weeks after mild biliary pancreatitis detected in our systematic review [17]. In the early laparoscopic cholecystectomy group, the re-admission rate (primary endpoint) during 6 months follow-up is set at 1% since, similar to the interval group, patients may be re-admitted for recurrent biliary events.

To demonstrate a reduction of the primary endpoint from 8.2 to 1.0% with power 80% and two-sided alpha of 5%, 132 patients will have to be included in each study group (PS Calculations, version 2.1.3; Vanderbilt University, Nashville, TN, USA). Taking a 0.5% (*n* = 2) loss to follow-up (based on the previous PROPATRIA and PANTER trials [38,39]), a total of 266 patients will have to be randomized.

#### Descriptive statistics

For dichotomous data, frequencies will be presented. Continuous data will be presented as mean and standard deviation or median and interquartile range. Baseline characteristics (all prior to randomization) are: age, sex, body mass index, etiology of pancreatitis, ASA classification, co-morbidity (that is, cardiovascular disease, pulmonary disease, chronic renal insufficiency or diabetes), predicted severity (Acute Physiology and Chronic Health Evaluation II score, Imrie score and C-reactive protein), time from onset of symptoms to randomization, and time from onset of symptoms to cholecystectomy.

#### Analyses

All analyses will be by intention-to-treat, meaning that all randomized patients are included in their initially assigned study arm, regardless of adherence to study protocol. There will be a blinded outcome assessment after the last patient in the trial has completed the follow-up. An adjudication committee blinded for treatment allocation will evaluate each patient using the raw data for the possible occurrence of the primary endpoint. Disagreements will be resolved in a plenary consensus meeting.

The primary endpoint will be compared between the treatment groups and subsequent analyses are directed at the secondary and safety endpoints. To compare the safety and efficacy of both treatment strategies, all individual endpoints of the combined primary, secondary and safety endpoints will be taken together and compared between both treatment groups.

Results are presented as risk ratios with corresponding 95% confidence intervals. Two-tailed *P* <0.05 is considered statistically significant with correction for multiple testing. In the event of imbalance between the two treatment groups a multivariable logistic regression will be used to correct for possible confounders. A *post-hoc* analysis will compare cholecystectomies with and without cholangiography.

#### Additional analyses

A predefined subgroup analysis will be performed on patients with or without ES, per center and age (<75 years vs. >75 years). We will use logistic regression models to perform a formal test for interaction to assess whether treatment effects differed significantly between these subgroups.

The costs of both treatment strategies for the whole follow-up period of 6 months will be compared. All costs will be estimated based on the actual input in terms of resource use, personnel and indirect costs from loss of productivity due to sick leave (assessed by the Health and Labour Questionnaire).

#### Premature termination of the study

In addition to assessing the safety of the patients in the study, the DSMC will check for early proof of efficacy during an interim analysis for benefit, which will be performed halfway through the study. The Peto approach will be followed, meaning that the study will only be stopped for beneficial effects in the event of *P* <0.001 [40]. The trial will not be stopped for futility, because this is the first randomized trial on this subject and treatment policy worldwide will be influenced by the results, regardless of the outcome.

If the DSMC suspects harm there will be a meeting between the DSMC, the steering committee and an independent statistician. During this meeting there should be a discussion about any potential causal relation between early cholecystectomy and harm based on the current literature.

The trial will be prematurely terminated if, after 4 years of inclusion, the required number of patients has not been reached, meaning a 60% delay in relation to the planned inclusion period of 2.5 years (30 months). Endpoints are not monitored sequentially.

#### Publication policy

All members of the steering committee and investigators involved in the daily logistics of the trial will be mentioned as an author.

For others, co-authorship will be based on the international guidelines, with a maximum of one co-author per participating site. Participating clinicians that do not fulfill these criteria will be listed as a collaborator and the journal will be asked to present the names of all collaborators to be listed in PubMed.

The order of authors will be based primarily on scientific input and secondarily on the number of randomized patients.

## Discussion

The PONCHO trial is designed to answer the question of whether early cholecystectomy leads to a reduction of re-admissions for biliary events in patients with a first episode of mild biliary pancreatitis.

Several treatment guidelines state that cholecystectomy should be performed in the first weeks after recovery of mild biliary pancreatitis in order to minimize re-admissions for biliary events [4,6,7,10,11,16]. In a systematic review we demonstrated that cholecystectomy should probably be performed during index admission because an early procedure was not associated with an increased risk of complications whereas interval cholecystectomy (after median 40 days) was associated with a biliary event recurrence rate of 18% [17]. In contrast to this finding, several nationwide audits from the UK, the USA, Germany and Italy have shown that most patients undergo cholecystectomy weeks or even months after discharge from the hospital for mild biliary pancreatitis [12-15]. As long as the gallbladder is *in situ*, these patients are at increased risk for re-admissions for biliary events including a potentially fatal episode of acute pancreatitis or other biliary events.

Why are surgeons not routinely performing early laparoscopic cholecystectomy after biliary pancreatitis? Early cholecystectomy may have three potential downsides: a technically more difficult and demanding procedure potentially resulting in more complications; poorer patient condition; and logistical hurdles.

Traditionally, early cholecystectomy has been suggested to be technically more demanding than interval cholecystectomy but data to support this statement are lacking. Notably, a recent study found that early cholecystectomy was technically less demanding, which is in keeping with the nature of peritoneal healing and adhesion formation [41]. This concept is supported by a recent retrospective study from India that focused on difficult dissection during laparoscopic cholecystectomy after mild biliary pancreatitis [18].

Traditionally, it is felt that patients should recover fully from pancreatitis prior to cholecystectomy being performed. However, the current study will only include patients with mild pancreatitis who are fit to undergo surgery. In contrast to severe pancreatitis, patients with mild pancreatitis recover quickly, and are discharged within 5 to 10 days after admission.

Problems with operating room capacity could arise due to the need for semi-urgent (<72 hours) cholecystectomy. In the participating centers, however, dedicated operating room programs for semi-urgent surgery are present, and hence no major problems with protocol compliance are envisaged [41.

The only way to provide convincing, level I evidence that early cholecystectomy is indeed superior to interval cholecystectomy is to perform a randomized controlled trial. A double-blinded controlled trial would be the optimal design. However, due to the difference in timing of cholecystectomy, blinding is not possible. To compensate for this fact there will be a blinded outcome assessment.

A time interval of 72 hours was chosen for the early cholecystectomy group to provide a feasible time frame for semi-urgent cholecystectomy by an experienced surgeon. The 25-day to 30-day interval (4 weeks) was chosen as a trade-off between: the Dutch guideline that advices cholecystectomy within 3 weeks after discharge, and the data from our retrospective multicenter study demonstrating that cholecystectomy is delayed for 6 weeks in current Dutch clinical practice [16].

The rationale for including ASA I and II older people/octogenarians is because several series have demonstrated that cholecystectomy is safe, even after ES, in older patients [42,43]. Patients with severe pancreatitis are excluded because this is considered an indication for delayed cholecystectomy [9].

For the proper timing of randomization we have chosen five discharge criteria that all have to be fulfilled in order to only include patients who are fit to undergo surgery and are without signs of severe pancreatitis. In contrast to severe pancreatitis, patients with mild pancreatitis recover quickly and are typically discharged within 5 to 10 days. There will be a variation in time since the onset of pancreatitis and hospital admission and between admission and discharge. This variation is mainly caused by co-morbidity. Owing to the randomization, there should be no relevant differences between both study arms.

The primary endpoint is a composite endpoint of mortality and re-admissions for biliary events. This composite endpoint was chosen because a study aimed at demonstrating a reduction in mortality only would require an unrealistic large sample size. In addition, other studies have shown that re-admissions for biliary events have much impact on the prognosis of patients [16].

The PONCHO trial is a randomized controlled multicenter trial designed to show a reduction in the composite primary endpoint of re-admissions for biliary events and mortality following an early cholecystectomy compared with an interval cholecystectomy in patients with a first episode of mild biliary pancreatitis.

### Trial status

The trial was registered in the ISRCTN register on 29 June 2010. The first patient was randomized on 22 December 2010. As of 21 October 2012, 172 patients have been randomized and inclusion is on schedule.

## Abbreviations

ASA: American Society of Anesthesiologists; CECT: contrast-enhanced Computed Tomography; DSMC: Data and Safety Monitoring Committee; ERCP: endoscopic retrograde cholangiopancreaticography; ES: endoscopic sphincterotomy; ISRCTN: International Standard Randomised Controlled Trial Number; PONCHO: Pancreatitis of biliary origin, optimal timing of cholecystectomy.

## Competing interests

The authors declare that they have no competing interests.

## Authors’ contributions

SAB drafted the manuscript. MGB, SvB, OJB, HCvS, HGG, BvR and DB co-authored the writing of the manuscript. MGB, SAB, OJB, HCvS, AFS, VBN, BJW, MAB, MJB, RT, BLW, HGG, BvR and DB participated in the design of the study during several meetings of the Dutch Pancreatitis Study Group. MGB and DB performed the sample size calculation. All authors critically assessed the study design or included patients in the study, edited the manuscript, and read and approved the final manuscript.

## Authors’ information

Steering committee: BL Weusten, R Timmer, MGH Besselink (also Academic Medical Center Amsterdam), B van Ramshorst, D Boerma (chair), St Antonius Hospital; HG Gooszen, SAW Bouwense, Radboud University Nijmegen Medical Centre; OJ Bakker, HC van Santvoort, University Medical Center Utrecht; AFM Schaapherder, Leiden University Medical Center; VB Nieuwenhuijs, University Medical Center Groningen; BJM Witteman, Hospital Gelderse Vallei Ede; MA Brink, Meander Medical Center; and MJ Bruno, Erasmus Medical Center.

Clinical centers (principal investigators) in the Netherlands (alphabetical order): Academic Medical Center, Amsterdam (MA Boermeester); Canisius-Wilhelmina Hospital, Nijmegen (C Rosman); Catharina Hospital, Eindhoven (IHJT de Hingh); Erasmus Medical Center, Rotterdam (CH van Eijck); Gelre Hospital, Apeldoorn (P van Duijvendijk); Hospital Gelderse Vallei, Ede (Ph Kruyt) MD; Jeroen Bosch Hospital, Den Bosch (K Bosscha); Leiden University Medical Center, Leiden (AFM Schaapherder); Maastricht University Medical Center, Maastricht (CHC Dejong); Meander Medical Center, Amersfoort (EC Consten); Medical Spectre Twente, Enschede (JJGM Gerritsen); Radboud University Nijmegen Medical Center, Nijmegen (H van Goor); Reinier de Graaf Gasthuis, Delft (JJ Scheepers); Rijnstate Hospital, Arnhem (MB Spanier); St Antonius Hospital, Nieuwegein (D Boerma); St Elisabeth Hospital, Tilburg (J Heisterkamp); University Medical Center Groningen, Groningen (VB Nieuwenhuijs); and University Medical Center Utrecht, Utrecht (IQ Molenaar).

Key staff at coordinating centers: St Antonius Hospital: MGH Besselink (also University Medical Center Utrecht and Academic Medical Center), B van Ramshorst and D Boerma (principal investigator); Radboud University Nijmegen Medical Centre: HG Gooszen, SAW Bouwense (study coordinator).

DSMC: C Mulder, VU University Medical Center (chair); MA Cuesta, VU University Medical Center; AL Verbeek, Radboud University Nijmegen Medical Centre; AC Vahl, Onze Lieve Vrouwe Gasthuis.

Independent physician: AJPM Smout, Academic Medical Center, Amsterdam.
